# Anatomical Study of the Triangle of Brocq and Mouchet and Its Clinical Implications for Coronary Interventions in Human Cadaveric Hearts

**DOI:** 10.7759/cureus.112440

**Published:** 2026-07-11

**Authors:** Aarthi V, Kalpana Ramachandran

**Affiliations:** 1 Department of Anatomy, Sri Ramachandra Institute of Higher Education and Research, Chennai, IND

**Keywords:** circumflex artery, coronary anatomy, great cardiac vein, left anterior descending artery, triangle of brocq and mouchet

## Abstract

Background: The triangle of Brocq and Mouchet is a clinically significant arteriovenous region on the anterior surface of the heart, formed by the anterior interventricular artery (AIV), circumflex artery (Cx), and great cardiac vein (GCV). Despite its relevance in cardiac surgery and interventional cardiology, detailed anatomical characterization of this region remains limited.

Objective: This study aimed to evaluate the presence, morphological variations, and vascular relationships of the triangle of Brocq and Mouchet in human cadaveric hearts, with emphasis on its clinical significance.

Materials and methods: A descriptive cadaveric study was conducted on 30 adult human hearts obtained from routine anatomical dissections at Sri Ramachandra Institute of Higher Education and Research (Deemed to be University) (SRIHER (DU)). Specimens with intact coronary vasculature were included. Detailed microdissection was performed to identify the LAD, Cx, and GCV. The arteriovenous triangle was first assessed for its presence and morphology. Based on the anatomical configuration formed by the AIV, Cx, and GCV, the triangle was classified into five morphological types: closed, inferiorly open, superiorly open, completely open, and absent. The relationship of the GCV with the LAD and Cx was recorded as superficial, deep, crossing, or parallel. Additional arterial components, including median and diagonal branches, were documented.

Results: The triangle was present in 28 (93.3 %) specimens. The closed type was the most frequent pattern (15 (50%) specimens), followed by the inferiorly open type (12 (40%) specimens), the superiorly open type (1 (3.3%) specimen), and absence of the triangle (2 (6.7%) specimens). No completely open type was observed. The GCV was predominantly deep to the LAD (16 (59.3%) specimens) and superficial to the Cx (21 (72.4%) specimens), with mixed relationships observed in 15 (50%) specimens. Additional arterial components were identified as the median artery in 11 (39.3%) specimens and diagonal branches in 8 (28.6%) specimens, with most structures located superficial to the GCV in 17 (60.7%) specimens.

Conclusion: The triangle of Brocq and Mouchet showed high prevalence and considerable anatomical variability in morphology and vascular relationships. These variations have important implications for coronary interventions, including catheterization, coronary artery bypass grafting (CABG), and electrophysiological procedures. A comprehensive anatomical understanding of this region is essential for improving procedural safety, minimizing iatrogenic complications, and supporting better clinical outcomes.

## Introduction

The coronary arterial system is a complex anatomical and physiological network essential for the maintenance of myocardial perfusion and normal cardiac function. The triangle of Brocq and Mouchet is an important arteriovenous landmark on the anterior (sternocostal) surface of the heart, but it has not been well reported in the anatomical literature [1,2]. According to Federative International Programme for Anatomical Terminology (FIPAT), the lateral boundaries of this triangle are formed by the anterior interventricular branch (left anterior descending artery (LAD)) and the circumflex branch (LCx) of the left coronary artery, while its base is formed by the great cardiac vein (GCV) [3]. It is located between the conus arteriosus and the left atrium in the body. This region is commonly encountered during cardiological interventions and cardiac surgical procedures; therefore, a detailed understanding of its anatomical features is essential [4].

The triangle of Brocq and Mouchet was originally described by the French anatomists Brocq and Mouchet in their studies of the coronary vasculature and has since been recognized as an important arteriovenous anatomical landmark on the anterior surface of the heart [5]. Despite its anatomical and clinical significance, the morphology and variations of this triangle remain relatively underreported in the contemporary literature [6]. The morphology of the triangle of Brocq and Mouchet is variable and is determined primarily by the relationship of the great cardiac vein (GCV) to the arterial boundaries formed by the anterior interventricular branch (left anterior descending artery (LAD)) and the circumflex branch (Cx) of the left coronary artery. In the present study, the triangle was classified morphologically into five types: closed, inferiorly open, superiorly open, completely open, and absent [7]. A closed triangle is formed when the GCV crosses both arterial boundaries, whereas variations in the course of the GCV may result in inferiorly open, superiorly open, or completely open configurations. In some specimens, the convergence of the constituent vessels is absent, resulting in the absence of the triangle [3]. These morphological variations are clinically important because they alter the spatial relationships of the coronary vessels and may influence diagnostic, interventional, and surgical cardiac procedures. In rare instances, the triangle may be absent altogether due to a lack of convergence between the associated vessels. These morphological variations may affect the anatomical relationships of the coronary vasculature and have important implications for diagnostic and interventional cardiac procedures [5].

Other arteries may also run in the triangle of Brocq and Mouchet, such as the diagonal branches of the left coronary system and the median artery [8]. If present, the median artery is a third branch of the LCA, where it terminates near the apex of the triangle. Typically, it involves the vascularization of the anterior interventricular septum and left ventricular myocardium [9]. Branches of the LAD or Cx may traverse the triangle and run either superficially or deep to the GCV, adding to the complexity of the area's vascular architecture [10]. The vascular arrangement within the triangle of Brocq and Mouchet may also reflect developmental variations in coronary artery formation. Coronary vessels develop from a primitive subepicardial vascular plexus that undergoes extensive remodeling during embryogenesis [11]. Variations such as the persistence of a median artery or additional diagonal branches may represent differences in the regression and persistence of embryonic vascular channels. Understanding these developmental aspects may provide additional insight into the anatomical diversity encountered within this region.

These anatomical variations have clinical significance, especially when using percutaneous coronary interventions (PCIs), coronary artery bypass grafting (CABG), placements of left ventricular leads, and mitral valve repairs. However, other vascular branches may be present in the triangle and could result in unwanted vascular injury during these procedures, which may cause postoperative complications, including arrhythmia, myocardial ischemia, or other adverse events [12]. The triangle of Brocq and Mouchet is an important transition area of the left coronary circulation where significant variations are seen in the spatial geometry of both the arterial and venous sides [13]. This area has complex three-dimensional relationships between the LAD artery, Cx artery, and GCV, which is characterized by the intersection and angular displacement of these three arteries [14]. This geometric variation leads to anatomical variation in this region and places special demands on the technical aspects of diagnosis or intervention in this region. The clinical relevance of the triangle of Brocq and Mouchet extends beyond its anatomical configuration, as variations in its morphology may directly affect the planning and execution of diagnostic and interventional cardiac procedures. [15]. Good anatomical knowledge of the cardiac morphology and the relationship of vessels in this area is imperative for better safety and efficacy of interventional cardiology and cardiac surgical procedures.

This triangle is of surgical and interventional importance, but its morphological variation has not been well documented in many populations. Previous cadaveric studies show that there is a great regional variability in both frequency and type of this triangle, thus highlighting the value of local anatomical data to optimize the success of the procedure and decrease the risk during the pre-procedure phase. In the present cadaveric study, the authors describe the triangle of Brocq and Mouchet in terms of its presence, type, vascular content, and its relationship with the LAD and Cx arteries. The purpose of this study is to improve the anatomical understanding of this clinically important trigone, which is necessary to perform safe and effective cardiac and surgical procedures.

Objectives of the study

This study aimed to investigate the anatomical variations of coronary vessels within the arteriovenous trigone of Brocq and Mouchet by assessing its presence in adult human cadaveric hearts; classifying its morphological types as closed, inferiorly open, superiorly open, completely open, or absent; analyzing the relationship of the great cardiac vein (GCV) with the anterior interventricular branch, commonly referred to as the left anterior descending artery (LAD), and the circumflex branch (Cx) of the left coronary artery as superficial, deep, or mixed; identifying additional arterial components within the triangle, including the ramus intermedius/median artery and diagonal branches; and describing the clinical relevance of these anatomical variations for coronary interventions and cardiac surgical procedures.

## Materials and methods

Study design and ethical consideration

**Figure 1:**
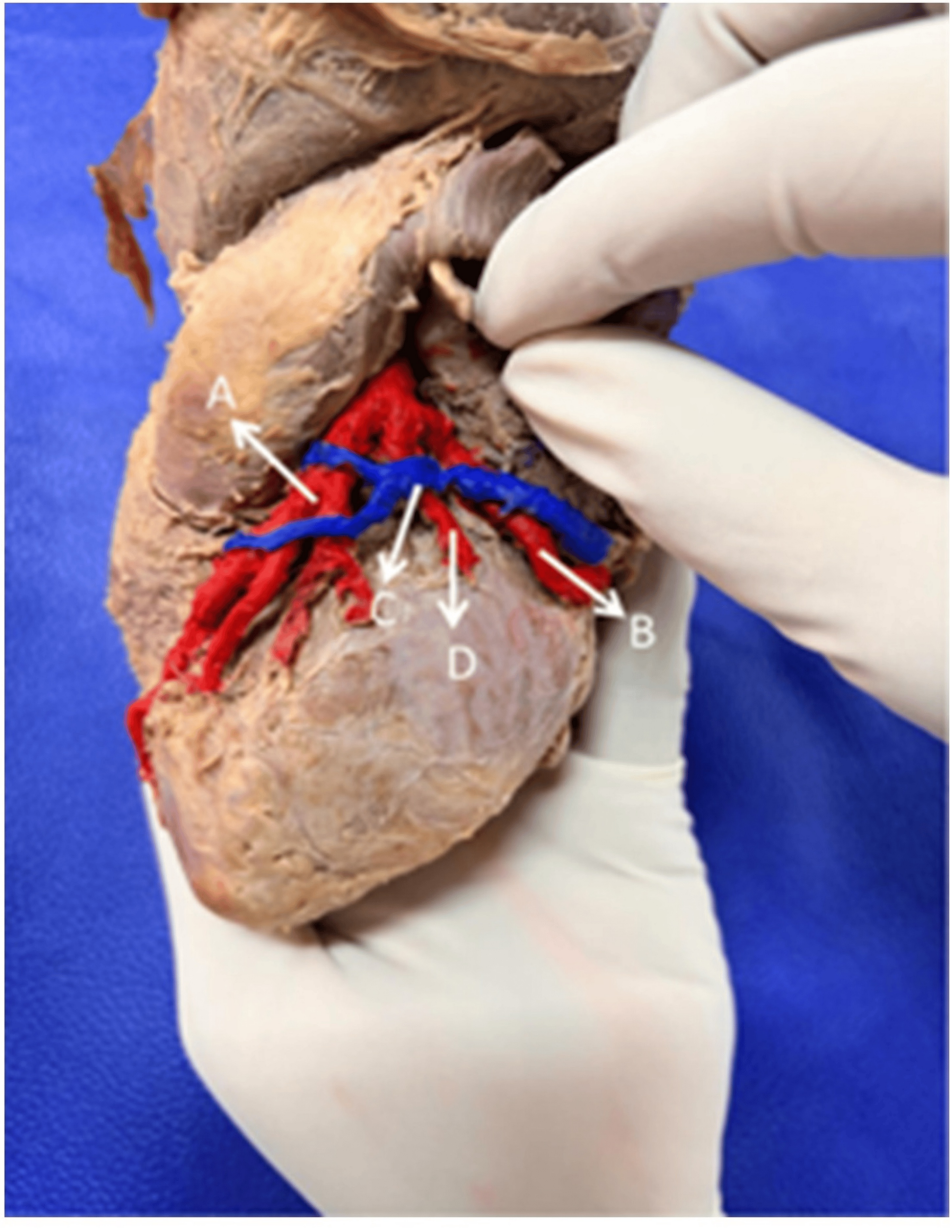
Representative cadaveric heart showing the closed triangle of Brocq and Mouchet A: left anterior descending artery, B: left circumflex artery, C: great cardiac vein, D: diagonal artery

This descriptive cadaveric study aimed to assess the anatomical morphology and vascular relationships of the triangle of Brocq and Mouchet in the adult human heart. The study was carried out in the Department of Anatomy, Sri Ramachandra Institute of Higher Education and Research (Deemed to be University) (SRIHER (DU)), a medical college attached to a tertiary care hospital. The cadaveric specimens were collected from routine undergraduate dissections of the thorax. The study was conducted after obtaining a waiver of consent from the Institutional Ethics Committee (approval number: CSP-MED/24/OCT/110/347), as the study involved preserved cadaveric material for academic and research studies. Standard anatomical dissection techniques were used in all specimens, and the handling of cadaveric material and the ethical requirements of the institution were respected. The study followed a uniform gross anatomical dissection protocol for all specimens to ensure consistency in specimen preparation, vessel identification, and morphological classification. A representative closed configuration of the triangle of Brocq and Mouchet, demonstrating the arteriovenous boundaries formed by the LAD, Cx, and GCV, is shown in Figure 1.

Sample selection and inclusion criteria

**Figure 2:**
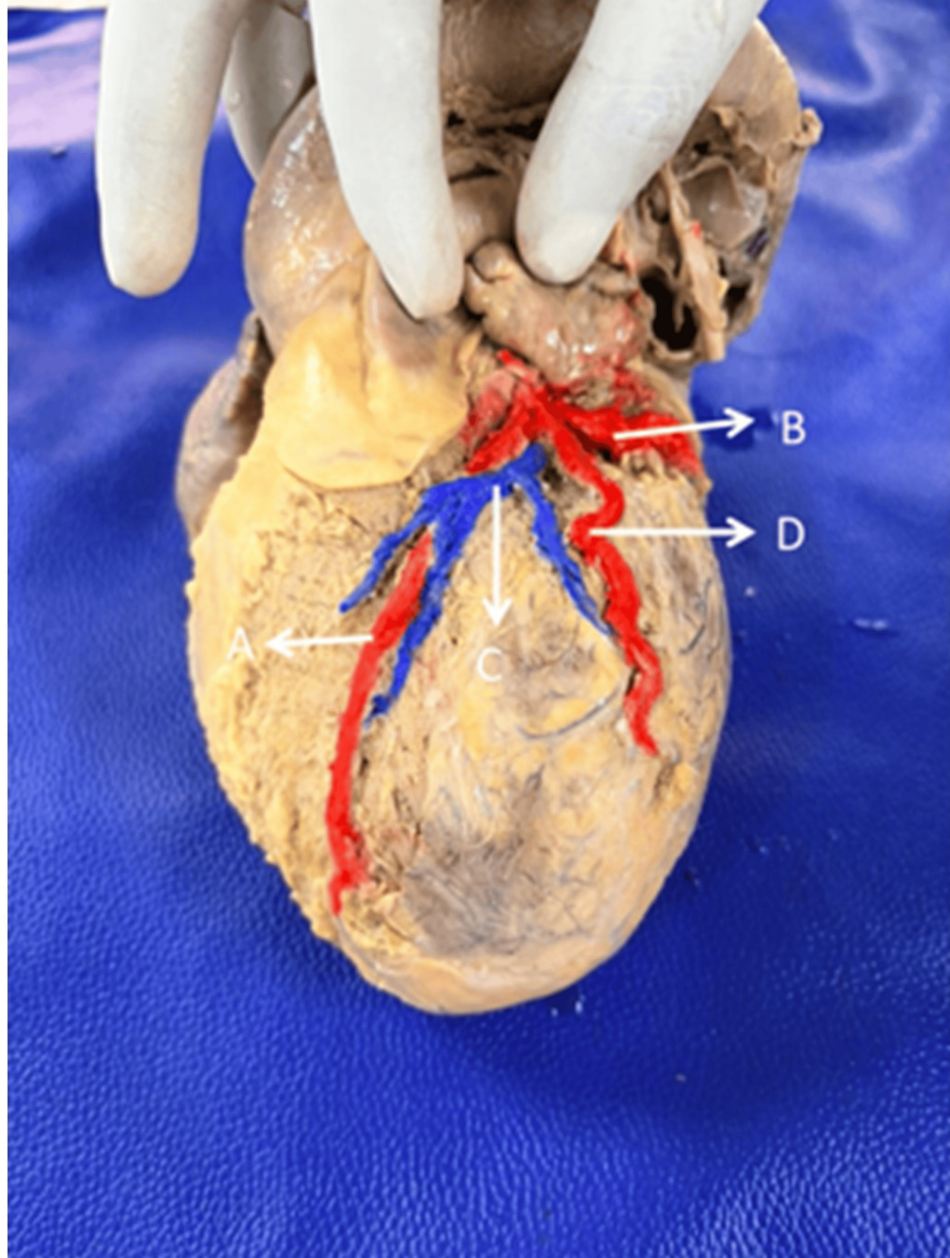
Cadaveric heart showing absence of the triangle of Brocq and Mouchet A: left anterior descending artery, B: left circumflex artery, C: great cardiac vein, D: diagonal artery

Thirty adult human cadaveric heart specimens were included in the study. This sample size was based on the availability of adult human cadaveric hearts with intact and well-preserved coronary vasculature during the study period. As this was a descriptive anatomical cadaveric study intended to document morphological variation rather than test a specific hypothesis, no formal sample size calculation was performed. Specimens with well-preserved and intact coronary vasculature were selected irrespective of donor age or sex. Age- and sex-related demographic data were not available for all specimens and, therefore, were not included in the analysis. Hearts with evidence of previous cardiac surgery, traumatic injury, gross anatomical distortion, or significant pathological changes were excluded from the study. During gross examination, coronary pathology or distortion was excluded when there was visible disruption, severe calcification, scarring, deformity, post-surgical alteration, traumatic damage, or loss of identifiable epicardial coronary vessels that prevented reliable tracing of the LAD, Cx, or GCV. This allowed the observed vascular patterns to be interpreted as anatomical variations rather than changes acquired through disease or previous procedures. All hearts were numbered sequentially before dissection to ensure specimen identification throughout the study. Information regarding age, sex, cause of death, and prior cardiovascular history was not available for the cadaveric specimens obtained from routine undergraduate dissection material. Consequently, specimen selection was based solely on anatomical preservation and integrity of the coronary vasculature. Figure 2 shows the convergence pattern of coronary vessels in which the typical triangular space is not formed due to an altered vascular arrangement.

Specimen preparation and dissection procedure

**Figure 3:**
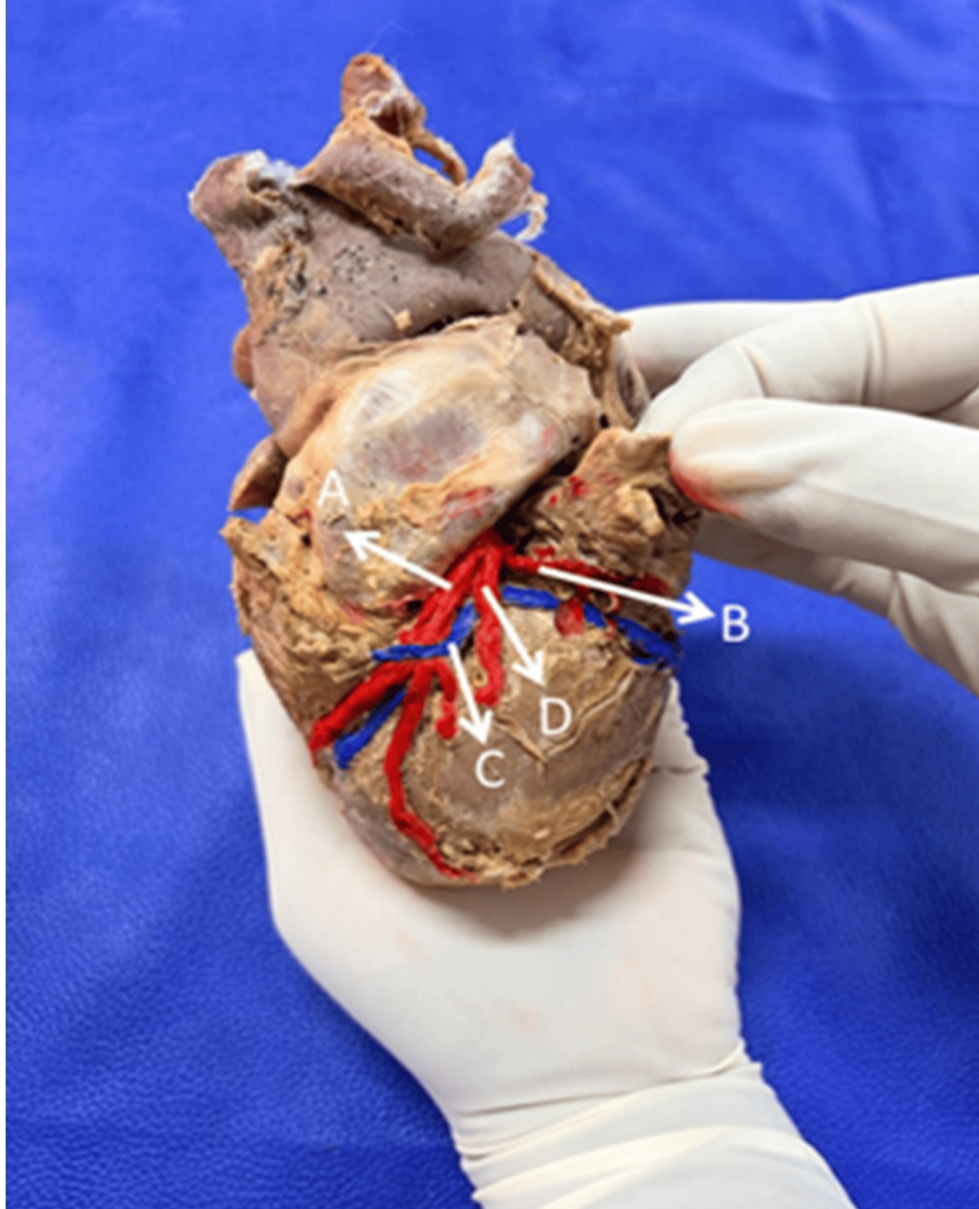
Superiorly open triangle of Brocq and Mouchet with labeled vessels A: left anterior descending artery, B: left circumflex artery, C: great cardiac vein, D: diagonal artery

After removal from the cadaver, each heart, along with a segment of the ascending aorta, was rinsed thoroughly and fixed in 10% formalin. The front of the heart was opened, and the heart was examined in detail. Superficial vessels were protected by careful removal of epicardial fat by standard microdissection. The left anterior descending artery and the circumflex artery were followed down their anatomical grooves, and the great cardiac vein was followed from the apex to the coronary sinus. The region of the arteriovenous connections where these vessels formed or failed to form the triangle of Brocq and Mouchet was particularly studied. The anterior interventricular branch, commonly referred to as the left anterior descending artery, the circumflex branch of the left coronary artery, and the great cardiac vein were used as the primary anatomical landmarks for identifying the triangle. Figure 3 shows the superiorly open variant, demonstrating the spatial relationship between the GCV and the left coronary arterial branches.

Anatomical assessment and classification

**Figure 4:**
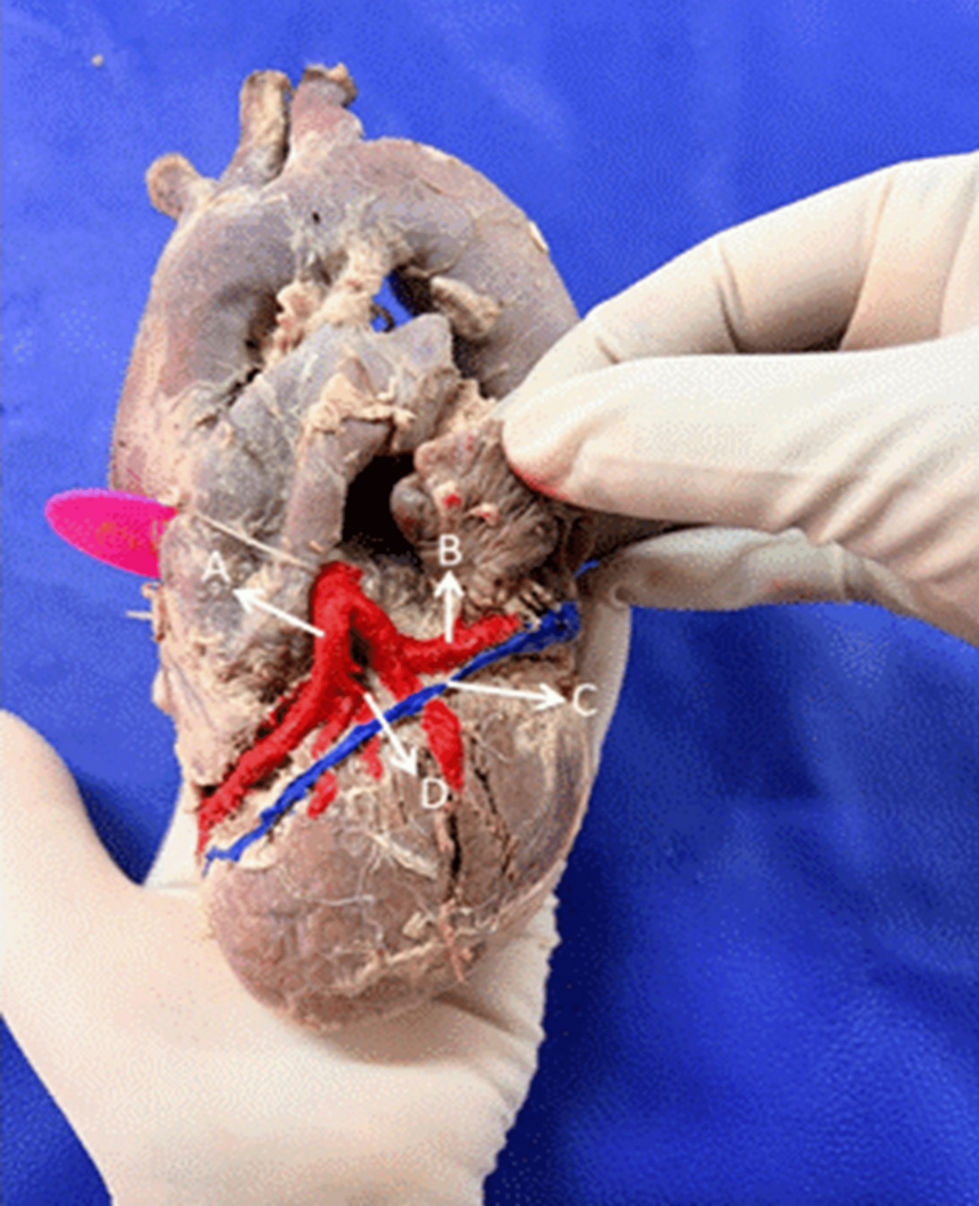
Inferiorly open triangle of Brocq and Mouchet with labelled vessels A: left anterior descending artery, B: left circumflex artery, C: great cardiac vein, D: diagonal artery

The presence or absence of the triangle of Brocq and Mouchet was evaluated in each specimen. When present, the triangle was classified according to its morphology as closed, superiorly open, inferiorly open, completely open, or absent. The closed type was defined when the GCV completed the vascular boundary of the triangle in relation to both arterial margins. The inferiorly open type was recorded when the lower part of the triangular space remained open, while the superiorly open type was recorded when the upper part of the triangular space remained open. The completely open type was defined as a configuration in which the spatial arrangement of the vessels did not complete a defined triangular boundary despite the presence of the constituent vessels. Absence was recorded when the constituent vessels did not form a recognizable triangular space. This classification was used because it describes the anatomical configuration of the triangle according to the spatial relationship between the GCV and the arterial boundaries, allowing consistent comparison of closed, open, and absent patterns. The anatomical relationship of the GCV with the LAD and Cx arteries was documented as superficial, deep, crossing, or parallel. For final reporting, the GCV relationship with each arterial branch was categorized as superficial or deep, and a mixed relationship was recorded when the GCV was superficial to one arterial branch and deep to the other. Additional arterial structures within the trigone, including median arteries and diagonal branches, were identified, and their relationship to the GCV was recorded. The median artery was considered equivalent to the ramus intermedius when it arose as an additional branch between the LAD and Cx arteries. Representative photographs were obtained to document the observed morphological patterns. These observations enabled a systematic assessment of the anatomical variability and vascular relationships within this clinically important coronary arteriovenous region. All anatomical observations and morphological classifications were independently assessed by two observers, and any disagreement was resolved by joint review and consensus. Figure 4 shows the inferiorly open configuration, highlighting the course of the GCV relative to the LAD and Cx arteries.

## Results

Frequency of the triangle of Brocq and Mouchet

For the adult human cadaveric hearts, the triangle of Brocq and Mouchet was found in 28 hearts out of 30 (93.3%). Two (6.7%) specimens did not have a triangle. The results of this discovery suggest that the triangle is a common anterior surface cardiac landmark. Because of its high prevalence, it is important for anatomical, surgical, and interventional purposes, where the left coronary artery and the great cardiac vein are encountered during clinical procedures. Table 1 shows the frequency distribution of the triangle of Brocq and Mouchet, indicating its presence in most cadaveric heart specimens.

**Table 1:** Frequency of Brocq and Mouchet triangle

Frequency of Brocq and Mouchet triangle	Frequency	Percentage
Present	28	93.3
Absent	2	6.7
Total	30	100

Morphological classification of the triangle

Among the 28 specimens in which the triangle of Brocq and Mouchet was present, the closed type was the most common morphological pattern, observed in 15 (53.6%) specimens. The inferiorly open type was identified in 12 (42.9%) specimens, while the superiorly open type was observed in 1 (3.6%) specimen. No completely open triangle was encountered. Morphological classification percentages were calculated using only the specimens in which the triangle was present (n = 28). Table 2 shows the morphological classification of the triangle of Brocq and Mouchet according to closed, open, and absent anatomical patterns.

**Table 2:** Classification of types of triangles

Classification	Frequency	Percentage
Type I: closed	15	53.6
Type II: inferiorly open	12	42.9
Type III: superiorly open	1	3.6
Type IV: completely open	0	0
Total	28	100

Relationship of the great cardiac vein with the LAD and Cx

The relationship of the great cardiac vein (GCV) with the left anterior descending artery (LAD) and circumflex artery (Cx) showed anatomical variability. Among the 28 specimens in which the triangle of Brocq and Mouchet was present, the GCV was superficial to the LAD in 12 (42.9%) specimens and deep to the LAD in 16 (57.1%) specimens. In relation to the Cx artery, the GCV was superficial in 20 (71.4%) specimens and deep in 8 (28.6%) specimens. A mixed relationship, defined as the GCV being superficial to one arterial branch and deep to the other, was observed in 12 (42.9%) specimens. Table 3 shows the anatomical relationship of the GCV with the LAD and Cx arteries.

**Table 3:** Relationship of the GCV with LAD and Cx arteries A mixed relationship (GCV superficial to one arterial branch and deep to the other) was observed in 12 (42.9%) specimens. This represents a combined pattern and is therefore reported separately from the individual LAD and Cx relationships.*Percentages were calculated based on the 28 specimens in which the triangle of Brocq and Mouchet was present. Mixed relationship indicates specimens showing variable anatomical relationships of the GCV with the LAD and Cx arteries. GCV: great cardiac vein, LAD: left anterior descending artery, Cx: circumflex artery, n: number of specimens/cases, %: percentage

Relationship of GCV	LAD (n (%))	Cx (n (%))
Superficial	12 (42.9)	20 (71.4)
Deep	16 (57.1)	8 (28.6)
Total	28 (100)	28 (100)

Contents of the triangle in relation to the great cardiac vein

The predominant feature of the contents of the triangle was arterial, and the relationships with the great cardiac vein were variable. Multiple vascular structures could be identified within the same specimen; therefore, the categories presented in Table 4 are not mutually exclusive in 11 (39.3%) specimens; the median artery was observed, five of which were superficial and six of which were deep to the GCV. The Cx was found in 8 specimens, and most of them were found superficial to the GCV. Eight specimens had the diagonal branches of both the LAD and Cx. In total, there were 17 superficial and 11 deep to the GCV, which further indicates there is great anatomical variation. Table 4 shows the contents of the triangle of Brocq and Mouchet in relation to their superficial or deep position to the great cardiac vein.

**Table 4:** Additional vascular structures observed within the triangle of Brocq and Mouchet and their relationship to the GCV Multiple vascular structures could occur within a single specimen; therefore, the categories are not mutually exclusive, and the total number of observations exceeds the total number of specimens studied (n = 28). Percentages were calculated using the 28 specimens in which the triangle of Brocq and Mouchet was present. GCV: great cardiac vein, Cx: circumflex artery, LAD: left anterior descending artery, n: number of specimens/cases, %: percentage

Contents of the triangle	Superficial to GCV (n (%))	Deep to GCV (n (%))	Total (n (%))
Median artery	5 (45.5)	6 (54.5)	11 (39.3)
Diagonal branch of Cx	6 (75)	2 (25)	8 (28.6)
Diagonal branches of LAD + Cx	5 (62.5)	3 (37.5)	8 (28.6)
Venous tributary	1 (100)	0 (0)	1 (3.6)
Total	17 (60.7)	11 (39.3)	28 (100)

Diagrammatic representation of morphological types

**Figure 5:**
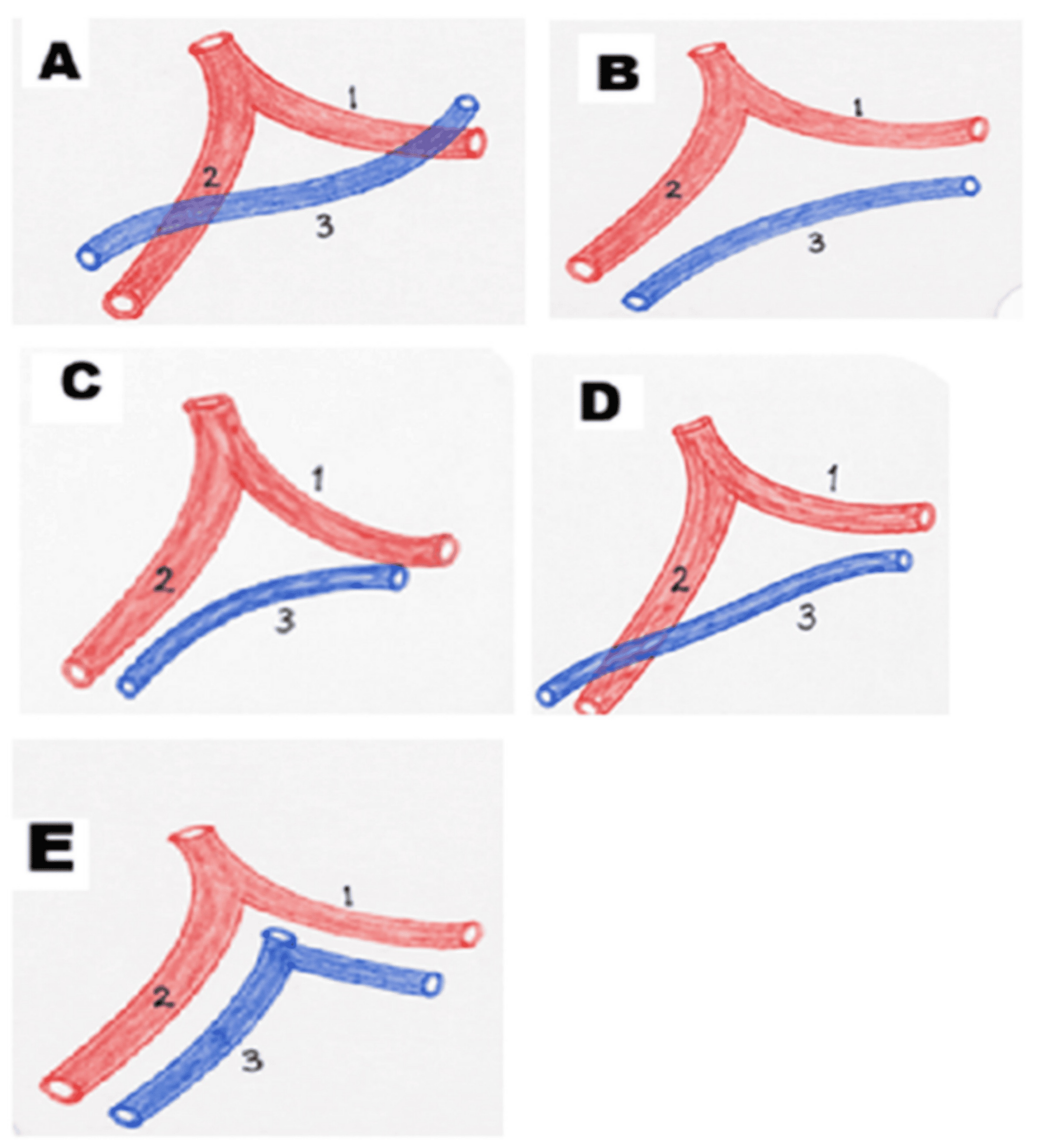
Diagrammatic representation of types of Brocq and Mouchet triangle A: type I, closed; B: type II, inferiorly open; C: type III, superiorly open; D: type IV, completely open; E: type V, absence of triangle; 1: Cx, circumflex artery; 2: LAD, left anterior descending artery; 3: GCV, great cardiac vein Source: Original hand-drawn illustration created by the authors

The diagram shows the main morphological variations of the triangle of Brocq and Mouchet according to the spatial relationship between the circumflex artery, left anterior descending artery, and great cardiac vein. The five configurations are represented as type I closed, type II inferiorly open, type III superiorly open, type IV completely open, and type V absence of the triangle. Figure 5 shows the schematic representation of the five morphological types of the triangle of Brocq and Mouchet with labelled coronary vessels.

## Discussion

Interventional relevance of the triangle of Brocq and Mouchet

Brocq and Mouchet's triangle is relevant in interventional cardiology in the context of coronary angiography, coronary catheterization, coronary sinus cannulation, and electrophysiological mapping. This area may have angulation and overlapping arteriovenous relationships that can cause technical challenges during catheter and guidewire navigation. A difference in the orientation of the great cardiac vein (GCV) in relation to the arterial boundaries may also contribute to the potential for unintentional vascular injury or poor device positioning. A thorough understanding of the anatomy in this area is therefore necessary for the safe planning and performance of diagnostic and therapeutic interventions to the heart.

Frequency of the triangle of Brocq and Mouchet

The present study found the triangle of Brocq and Mouchet in 28 (93.3%) specimens of the adult human cadaveric hearts, which means it is a frequent anatomical feature of the human heart. This prevalence is similar to that reported previously [4], which was 92.5%. A prevalence of 28 (93.3%) specimens was also recorded in another study, corroborating the regular occurrence of this triangle in studies of other populations [8]. These observations emphasize the significance of this region for coronary vascular evaluation, both anatomically and clinically [16,17].

Morphological variations in the triangle

The triangle of Brocq and Mouchet showed notable morphological variation among the studied specimens. The closed type was the most common configuration, followed by the inferiorly open type, while the superiorly open type was observed less frequently. These findings are consistent with previous studies in which the closed pattern was also reported as the predominant morphology [17]. Variability in the relationship between the great cardiac vein (GCV) and the left coronary arterial branches has also been described in earlier anatomical studies [8]. In contrast, some studies have reported predominance of the inferiorly open type, which may be related to methodological differences, including the use of corrosion cast techniques [16]. The completely open type was not observed in the present study, which may reflect population-specific anatomical variation, the limited sample size, or natural variation in the course of the GCV relative to the LAD and Cx arteries.

Relationship between the great cardiac vein and arterial branches

The left anterior descending artery (LAD) and circumflex artery (Cx) relationship with the GCV was variable; superficial, deep, crossing, and mixed were all seen. In the present study, the GCV was deeper into the LAD and superficial to the Cx. Another significant number of specimens also exhibited a mixed relationship. The results are in line with earlier reports of different disease courses depending on the coronary arterial branches [4]. The importance of deep positioning of GCV has also been highlighted as a potential problem during PCI and surgery [8,18].

Presence of additional arteries in the triangle

In the present study, other arterial structures in the triangle were also recorded, such as the median artery and diagonal branches. In 39.3% of the specimens, the median artery was identified as having a significant role in the study of the anatomical variants. The same observation was reported for the presence of the median artery in this trigone [17,18]. The interventricular septum and anterior ventricular wall may be supplied by the median artery. It could affect the surgical strategy during coronary artery bypass grafting and interventional procedures through the left coronary circulation.

Embryological significance

The occurrence of a median artery in 39.3% of specimens corroborates the idea of the persistence of embryonic vascular channels in the coronary circulation. During normal development, the coronary arteries develop from a primitive subepicardial vascular plexus that regresses and remodels. An extra-arterial channel could be an embryonic trifurcation of the left coronary artery. The presence of the diagonal branches in the triangle further strengthens the idea of variable regression of primitive vascular channels. The results suggest that the vascular complexity in this area may be a developmental difference in the coronary artery pattern.

Clinical implications

The results of the present study reveal the clinical significance of knowledge of the anatomy of the triangle of Brocq and Mouchet. The variations in the relationships of the GCV, LAD, and Cx, as well as accessorial arterial branches, can affect coronary catheterization, angiography, coronary artery bypass graft, left ventricular lead placement, and mitral valve repair. This triangle is also closer to the left fibrous ring and mitral valve apparatus, thereby adding to its surgical significance. Such detailed knowledge of these vascular relationships can contribute to minimizing iatrogenic injury, procedural accuracy, and cardiovascular surgical and interventional safety.

Limitation and future recommendation

In the present study, 30 adult human cadaveric hearts were examined; therefore, the findings may not be generalizable to larger and more diverse populations. The specimens were obtained from a single institution and were limited to cadaveric hearts with intact coronary vasculature. Age, sex, cause of death, cardiovascular history, and other clinical details were not available for the specimens, which prevented assessment of whether these factors influenced coronary vascular morphology. There was no radiological, angiographic, intraoperative, or clinical correlation; therefore, the findings should be interpreted as anatomical observations rather than direct evidence of procedural risk or clinical outcome. The study was based on gross anatomical dissection, and subtle vascular relationships may differ from those observed in live imaging or operative settings. The clinical implications described in this study require further validation through imaging-based, intraoperative, and larger multicenter studies.

Future research should include larger samples from diverse demographic and geographic populations to improve generalizability. Correlation with computed tomography coronary angiography, invasive coronary angiography, intraoperative observations, and clinical procedural outcomes would strengthen the translational relevance of these findings. Future studies should also assess age, sex, coronary dominance pattern, cardiovascular history, and associated coronary artery variants to better understand factors influencing the morphology of the triangle of Brocq and Mouchet. Three-dimensional reconstruction and imaging-based anatomical studies may further clarify the spatial relationship between the GCV, LAD, and Cx arteries and support pre-procedural planning for coronary interventions, CABG, electrophysiology procedures, and mitral valve surgery.

## Conclusions

This study provides significant anatomical information about the triangle of Brocq and Mouchet, which was identified in 28 of 30 cadaveric heart specimens (93.3%), with the closed configuration being the predominant morphological type. The findings demonstrate variability in the relationship of the great cardiac vein with the anterior interventricular branch, commonly referred to as the left anterior descending artery, and the circumflex branch of the left coronary artery, including superficial, deep, and mixed patterns. Additional vascular structures, including the ramus intermedius/median artery and diagonal branches, were also observed within the triangle. These findings support the anatomical importance of this region and may assist clinicians in anticipating vascular variation during coronary interventions, coronary artery bypass grafting, electrophysiological procedures, and related cardiac surgical planning. Since this was a cadaveric anatomical study, the clinical implications should be interpreted as potential procedural relevance rather than direct evidence of clinical outcomes.
